# Non-poissonian Distribution of Point Mutations in DNA

**DOI:** 10.3389/fchem.2020.00038

**Published:** 2020-01-31

**Authors:** Nigora Turaeva, Boris L. Oksengendler

**Affiliations:** ^1^Department of Biological Sciences, Webster University, Saint Louis, MO, United States; ^2^Arifov Institute of ion-plasma and Laser Technologies, Tashkent, Uzbekistan; ^3^Ural Federal University, Yekaterinburg, Russia

**Keywords:** Löwdin's mechanism, spontaneous mutation, non-poissonian distribution function, double proton transfer, autocatalytic reaction

## Abstract

In general, for chemical reactions occurring in systems, where fluctuations are not negligibly small, it is necessary to introduce a master equation for distribution of probability of fluctuations. It has been established that the monomolecular reactions of a type as *A* ↔ *X* are described by the master equation, which leads to a Poisson distribution with the variance equal to the average value *N*_0_. However, the consideration of the Löwdin mechanism as autocatalytic non-linear chemical reactions such as *A* + *X* ↔ 2*X* and the corresponding master equation lead to a non-Poissonian probability distribution of fluctuations. In the presented work, first-order autocatalysis has been applied to the Löwdin's mechanism of spontaneous mutations in DNA. Describing double proton transfers between complimentary nucleotide bases along the chain by first-order autocatalytic reactions, the corresponding master equation for protons in tautomeric states becomes non-linear, and at non-equilibrium conditions this leads to the non-Poissonian distribution of spontaneous mutations in DNA. It is also suggested that the accumulation of large fluctuations of successive cooperative concerted protons along the chain may produce higher non-linearities which could have a significant impact on some biochemical processes, occurring in DNA.

## Introduction

**Figure 1:**
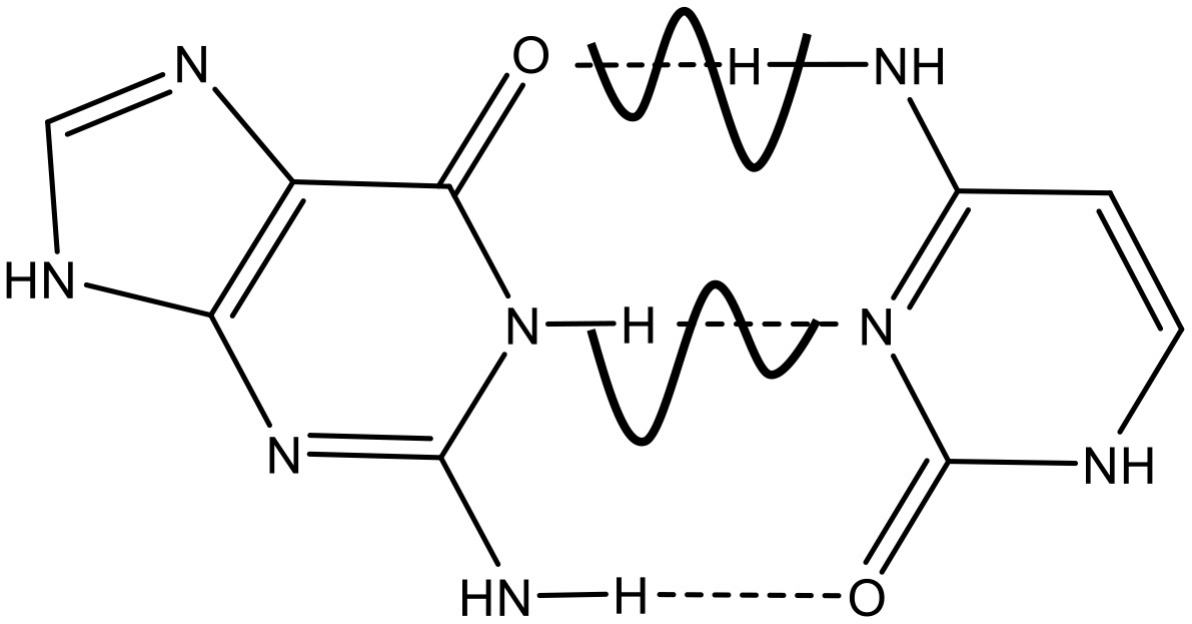
G-C → G* − C*: DPT in the double potential wells.

The role of mutations in DNA is crucial for human aging, metabolic and degenerative disorders and cancer, as well as for biological evolution of living systems (Löwdin, 1966; Friedberg et al., 2006). The point mutations caused by the substitution of one nucleotide base for another may occur during DNA replication by DNA polymerases, the performance of which is very important for genome integrity and transmission of genetic information in all living organisms. Although DNA replicates with high fidelity, DNA polymerase can make mistakes with the average frequency in the range of (10^−7^–10^−9^) per base pair per cell division (Drake et al., 1998). Spontaneous mutations are point mutations caused by the substitution of one nucleotide base of DNA for another one occurring due to endogenous factors during normal cell metabolism. The rare tautomeric hypothesis (Watson and Crick, 1953a,b; Topal and Fresco, 1976; Bebenek et al., 2011; Wang et al., 2011) originally proposed by J. Watson and F. Crick (Watson and Crick, 1953a,b) is considered as a possible mechanism of formation of spontaneous mutations suggesting the existence of different chemical forms of nucleotide bases, so called tautomers, in which the protons occupy in one of its tautomeric forms. The origination of mutagenic tautomers has not been completely established, although three possible mechanisms are discussed: (1) intramolecular single proton transfer in DNA bases (Basu et al., 2005; Gorb et al., 2005; Zhao et al., 2006; Brovarets and Hovorun, 2010, 2011; Brovarets et al., 2012); (2) proton transfer in a single base assisted by water molecules (Gorb and Leszczynski, 1998a,b; Kim et al., 2007; Fogarasi, 2008; Michalkova et al., 2008; Furmanchuk et al., 2011; Markova et al., 2017); (3) Löwdin's mechanism of double proton tunneling (DPT) between complimentary bases (Löwdin, 1963, 1966) (Figure 1).

Based on the rare tautomeric hypothesis by Watson-Crick, Löwdin (Löwdin, 1963, 1966) suggested that spontaneous mutations in DNA occur due to double proton transfer between two complementary bases along intermolecular H-bonds by quantum tunneling. Thus, each proton in the connecting hydrogen bonds can be in one of two quantum states, in deep or shallow potential wells. Following the pioneering works of Löwdin, the tautomeric base pairs (A^*^-T^*^, G^*^-C^*^) have been extensively studied in terms of their lifetime, the probability of occurrence and the energy by using different theoretical approaches (Florian et al., 1995; Florian and Leszczynski, 1996; Gorb et al., 2004; Villani, 2005, 2010; Ceron-Carrasco et al., 2009a,b; Brovarets et al., 2012), including DFT calculations, ab initio MP2, quantum mechanics. All these calculated findings substantially support the Watson-Crick's tautomeric hypothesis of the origin of spontaneous point mutations, constrained by thermodynamic and kinetic criteria (Florian et al., 1994; Dabkowska et al., 2005; Brovarets et al., 2012) to be relevant to spontaneous mutations. Based on the Löwdin's mechanism, the probability of formation of spontaneous mutations was calculated from the kinetics of double proton transfer during DNA replication by taking into account 2D Marcus theory of double proton transfer (DPT) (Turaeva and Brown-Kennerly, 2015). The model allows to establish the spontaneous mutation probability as a function of temperature, replication rate and solvent. It was also established that there are different factors impacting on DNA mutations, including external electric fields (Ceron-Carrasco et al., 2014), metallic cations (Ceron-Carrasco et al., 2012), even the genetic sequence (Ceron-Carrasco and Jacquemin, 2015) through the hydrogen transfers between the complimentary nucleotide bases in DNA. In the framework of Lowdin's mechanism of spontaneous mutation formation, we can suppose that those factors directly or indirectly change the potential barrier relief for proton transfers, leading to the change of the mutation rate and the distribution of mutations along the chain.

In the present work, we will show that the Löwdin's mechanism of spontaneous mutations results in a non-Poissonian distribution function of mutagenic tautomeric forms of DNA by using first-order autocatalysis. It is well-known that the first- and higher order autocatalysis was applied to different biological processes, including reproduction (Eigen, 1971; Biebricher et al., 1983; Schuster, 2018), cooperation (Higgs and Lehman, 2015; Schuster, 2018), chirality of biological molecules (Frank, 1953). The autocatalytic reaction of first order was applied to reproduction of RNA and DNA molecules (Biebricher et al., 1983), giving rise to their exponential growth with different reproducing variants leading to natural selection. The correct and error-prone RNA replication leading to point mutations was also described by the autocatalytic reaction of first order by introducing the mutation matrix with the assumption of uniform error rate (Eigen, 1971). Catalyzed replication leading to cooperation among replicators was described by the autocatalytic reaction of second order (Higgs and Lehman, 2015). In general, according to the evolution model (Schuster, 2018), the characteristic features of the first order autocatalysis include selection and optimization, while the second order autocatalysis covers oscillations, deterministic chaos, spontaneous pattern formation and high sensitivity to stochastic phenomena caused by small particle numbers. So, competitive reproduction gives rise to selection, but catalyzed reproduction is needed for cooperation of competitors.

In our model, the autocatalytic reaction of first order is applied to the process of double proton transfer in DNA, which gives a non-Poissonian distribution of tautomeric states of hydrogens along the chain which as a result of replication leads to spontaneous mutations.

The search of experiments on the distribution function of spontaneous mutations to verify our model leads to the Luria-Delbruck's experiments and distribution of mutants (Luria and Delbrück, 1943), that gives a non-Poissonian relationship for the distribution of mutant bacteria colonies consistent with the experimentally obtained values, in which the variance was considerably greater than the mean. We suppose that the non-Poissonian character of the distribution function of tautomeric forms of nucleotide bases can be counted toward the Löwdin's mechanism of origin of spontaneous mutations formation, since the intramolecular single proton transfer in DNA bases describing by the monomolecular reactions of a type as *A* ⇔ *X* is described by the master equation, leading to the Poissonian distribution, where the variance is equal to the average value *N*_0_.

## Stochastic Model of Spontaneous Mutation Formation

In general case, for chemical reactions occurring in systems, where fluctuations are not considered negligible small, it is necessary to introduce a master-equation for distribution of probability (Gardiner, 1983; Haken, 1983). Analyzing a monomolecular reaction of a type as *A* ⇔ *X* on the base of the master equation the Poisson distribution function with average value of *N*_0_ can be derived. It is well-known that at considering the reactions of *A* + *X* ⇔ 2*X* the corresponding kinetic equations for markovian processes become non-linear and this peculiarity leads to the non-Poissonian distribution functions. This result was proved by Nicolis and Prigogine (Nicolis and Prigogine, 1977) and caused a great scientific interest. The process of generation of spontaneous mutations in DNA through double proton transition during the replication can be treated as first-order autocatalytic chemical reactions, described by the following reaction scheme:

(1) $$\begin{array}{l} A\begin{array}{c} k_{1}\\ \Leftrightarrow \\ k_{-1} \end{array}X \end{array}$$

(2) $$\begin{array}{l} A+X\begin{array}{c} k_{2}\\ \Leftrightarrow \\ k_{-2} \end{array}2X \end{array}$$

**Figure 2:**
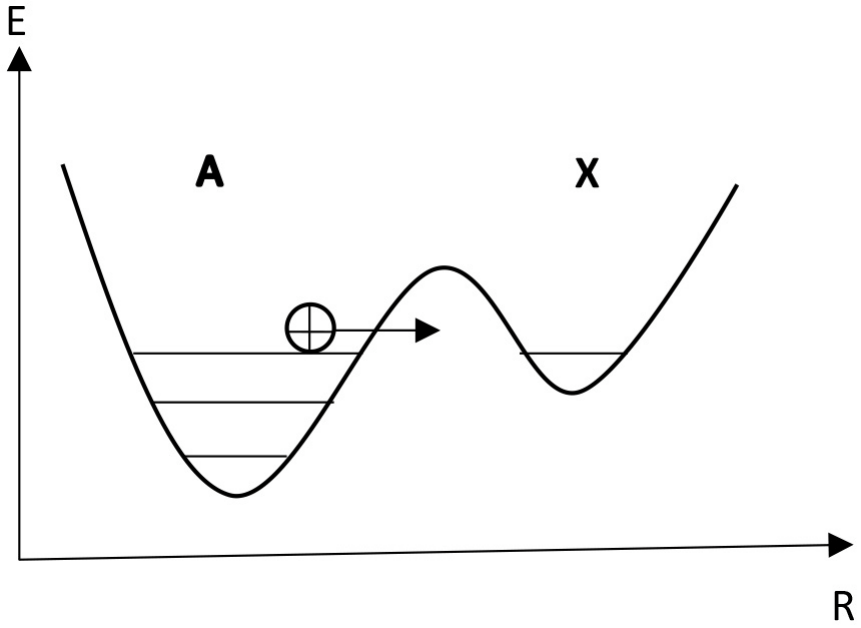
The double-well potential for a single proton tunneling.

Here A denotes protons along the DNA strand which are in their regular stable position, X is the protons in the tautomeric state (Figure 2), *k* and *k*_−_ are the rate constants of forward and reverse chemical reactions, respectively. The first reaction corresponds to the processes of the transfer of a proton from the regular position into the tautomeric state and vice versa, while the second reaction corresponds to the generation of tautomeric forms of nucleotide bases due to the interaction of the single proton in the tautomeric state with the regular proton and its relaxation into the single proton tautomeric state.

Denoting the number of protons in the tautomeric state by *N*, we can establish a master-equation for the distribution of fluctuations *P*(*N, t*) in the following general form:

(3) $$\begin{array}{l} \dot{P}\left(N,t\right)=w\left(N,N-1\right)P\left(N-1,t\right)\\ \;+\;w\left(N,N+1\right)P\left(N+1,t\right)-\{w\left(N+1,N\right)\\ \;+w\left(N-1,N\right)\}P\left(N,t\right). \end{array}$$

Here w is the transition probability per unit time. The crux of the master equation is to determine the transition rates explicitly for each chemical reaction. We investigate all transitions leading to N or going away from it. For spontaneous mutations in DNA we consider two types of transition—the tautomeric proton generation (*N* → *N* + 1, *N* – 1 → *N*) and the tautomeric proton annihilation (*N* + 1 → *N, N* → *N* − 1) for both chemical reactions. We will show in detail the derivation of total transition rate for the first reaction (1).

1. In the direction *k*_1_ (“birth” of a tautomeric proton X). The number of the transitions per second is equal to the occupational probability, *P*(*N, t*), multiplied by the transition probability per second, ω(*N* + 1, *N*), which is the product of the linear density of regular protons *N*_*A*_ along the DNA strand and the reaction rate *k*_1_. So, for transition → *N* + 1, we have *w*(*N* + 1, *N*) = *k*_1_*N*_*A*_*L*. Here *L* is the length of DNA. In the same way the transition rate is calculated for transition *N* − 1 → *N*: *w*(*N, N* − 1) = *k*_1_*N*_*A*_*L*.

For this reaction the total transition rate is received in the following form:

(4) $$\begin{array}{l} k_{1}N_{A}LP\left(N-1,t\right)-k_{1}N_{A}LP\left(N,t\right). \end{array}$$

2. In a similar way, we can find the reverse process of the first reaction. Here the number of tautomeric protons is decreased by 1 (“death” of a metastable proton X). Considering the “death” process for transitions *N* + 1 → *N* and *N* → *N* − 1, in the reverse *k*_ − 1_ direction (“death” of X) the total transition rate is equal to:

(5) $$\begin{array}{l} k_{-1}\frac{\left(N+1\right)}{L}LP\left(N+1,t\right)-k_{-1}\frac{N}{L}LP\left(N,t\right). \end{array}$$

3. For the reaction (2), in the *k*_2_ direction (“birth” of a tautomeric proton X), the total transition rate can be written:

(6) $$\begin{array}{l} k_{2}N_{A}\frac{N-1}{L}LP\left(N-1,t\right)-k_{2}N_{A}\frac{N}{L}LP\left(N,t\right). \end{array}$$

4. For the reaction (2) in the *k*_−2_ direction (“death” of a tautomeric proton X), the total transition rate can be written:

(7) $$\begin{array}{l} k_{-2}\frac{\left(N+1\right)N}{L^{2}}LP\left(N+1,t\right)-k_{-2}\frac{N\left(N-1\right)}{L^{2}}LP\left(N,t\right). \end{array}$$

Thus, by taking into account (Equations 4–7) the transition rates for the reactions (1) and (2) are given:

(8) $$\begin{array}{l} \omega \left(N,N-1\right)=k_{1}N_{A}L+k_{2}N_{A}\frac{N-1}{L}L; \end{array}$$

(9) $$\begin{array}{l} \omega \left(N,N+1\right)=k_{-1}\frac{\left(N+1\right)}{L}L+k_{-2}\frac{\left(N+1\right)N}{L^{2}}L; \end{array}$$

(10) $$\begin{array}{l} \omega \left(N+1,N\right)=k_{1}N_{A}L+k_{2}N_{A}\frac{N}{L}L; \end{array}$$

(11) $$\begin{array}{l} \omega \left(N-1,N\right)=k_{-1}\frac{N}{L}L+k_{-2}\frac{N\left(N-1\right)}{L^{2}}L. \end{array}$$

The stationary solution of the master equation (3) is determined as Eigen (1971):

(12) $$\begin{array}{l} P\left(N\right)=P\left(0\right)\overset{N-1}{\underset{n=0}{\prod }}\frac{\omega \left(n+1,n\right)}{\omega \left(n,n+1\right)}\\ \;=P\left(0\right)\overset{N-1}{\underset{n=0}{\prod }}\frac{k_{1}N_{A}L^{2}+k_{2}N_{A}nL}{k_{-1}\left(n+1\right)L+k_{-2}\left(n+1\right)n}. \end{array}$$

Let us denote the probabilities of two transitions by the following expressions:

(13) $$\begin{array}{l} \{\begin{array}{l} \omega \left(N,N-1\right)=\omega_{+}\left(N\right)\\ \omega \left(N,N+1\right)=\omega_{-}\left(N\right). \end{array} \end{array}$$

The condition of extremes of stationary *P*(*N*) is Haken (1983):

(14) $$\begin{array}{l} \frac{\omega_{+}\left(N_{0}+1\right)}{\omega_{-}\left(N_{0}\right)}=1. \end{array}$$

We can find the extreme solutions *N*_0_ of *P*(*N*) from the following expression which is received by rewriting (Equation 14) by taking into account Equations (13), (8), and (9):

(15) $$\begin{array}{l} \frac{k_{1}N_{A}L^{2}+k_{2}N_{A}N_{0}L}{k_{-1}\left(N_{0}+1\right)L+k_{-2}\left(N_{0}+1\right)N_{0}}=1. \end{array}$$

The solution of Equation (15) for *N*_0_ can be received:

(16) $$\begin{array}{l} N_{0}^{1,2}=\frac{-\left(k_{-2}+k_{-1}L-k_{2}N_{A}L\right)\pm \sqrt{{\left(k_{-2}+k_{-1}L-k_{2}N_{A}L\right)}^{2}-4k_{-2}\left(k_{-1}L-k_{1}N_{A}L^{2}\right)}}{2k_{-2}}. \end{array}$$

**Figure 3:**
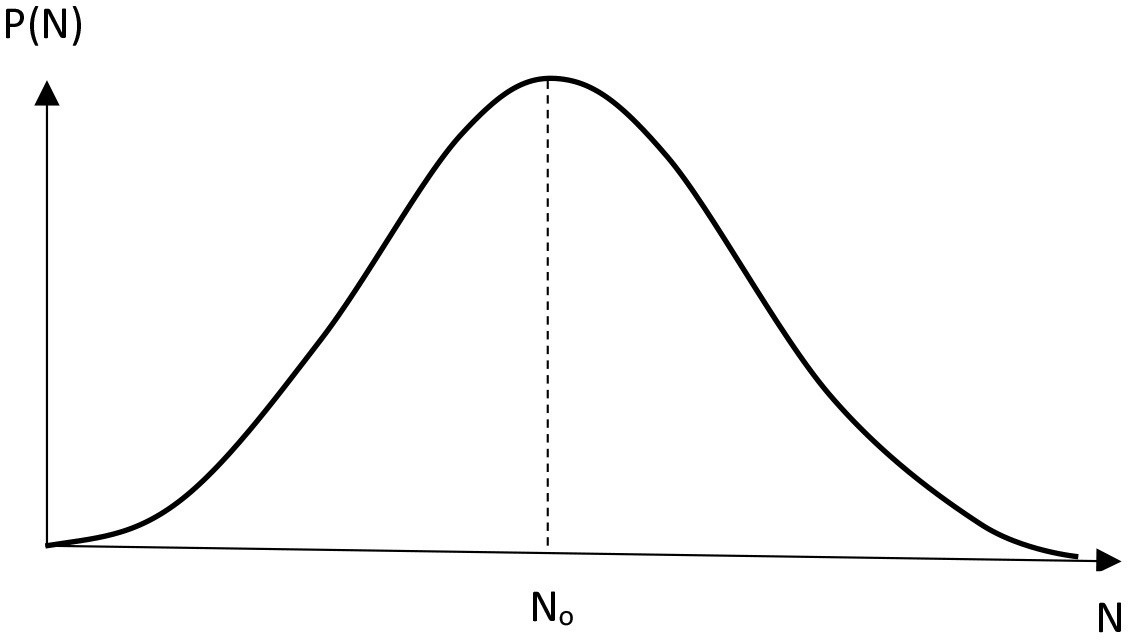
Non-poissonian distribution function P(N) of spontaneous mutations in DNA by Löwdin's mechanism.

The positive value of *N*_0_ can be obtained if *k*_ − 2_ + *k*_ − 1_*L* < *k*_2_*N*_*A*_*L*. The plot of the probability *P*(*N*) represents the curve with two maxima. By assuming that the transfer of the second proton takes place almost instantaneously compared to its reverse process, which corresponds to the large ratio of *k*_2_/*k*_−2_, we can receive the probability distribution function with one maximum (Figure 3):

(17) $$\begin{array}{l} N_{0}=\frac{k_{-1}-k_{1}N_{A}L}{k_{2}N_{A}-k_{-1}}. \end{array}$$

It is seen from Equation (17) that N_o_ is positive when the transfer of proton from the regular position into the tautomeric state proceeds slower than its reverse reaction (1), while the second proton is transferred almost instantaneously (2) compared to the reverse process of the first reaction (1).

If the principle of detailed balance is satisfied for both chemical reactions (1) and (2), then the Poisson distribution function for fluctuations can be deduced (Haken, 1983). We must write the detailed balance equations then for both reactions (1) and (2):

(18) $$\begin{array}{l} \omega_{1}\left(N,N-1\right)P\left(N-1\right)=\omega_{1}\left(N-1,N\right)P\left(N\right); \end{array}$$

(19) $$\begin{array}{l} \omega_{2}\left(N,N-1\right)P\left(N-1\right)=\omega_{2}\left(N-1,N\right)P\left(N\right). \end{array}$$

Dividing Equation (18) by Equation (19) and using the explicit expressions (Equations 8 and 11), we find the relation:

(20) $$\begin{array}{l} \frac{k_{1}N_{A}}{k_{-1}N/L}=\frac{k_{2}N_{A}}{k_{-2}N/L}\equiv \frac{\mu }{N}. \end{array}$$

Here μ is a constant. By using Equation (20) we can rewrite the detailed balance equation:

(21) $$\begin{array}{l} \omega \left(N,N-1\right)=\frac{\mu }{N}\omega \left(N-1,N\right). \end{array}$$

By inserting Equation (21) into Equation (12) at normalization condition of *P*(0) we find the Poisson distribution:

(22) $$\begin{array}{l} P\left(N\right)=\frac{\mu^{N}}{N!}e^{-\mu }. \end{array}$$

In general, however, for non-equilibrium processes, where the detailed balance principle is not valid, the character of distribution function *P*(*N*) (Equation 12) is non-Poissonian (Gardiner, 1983; Haken, 1983). Since double proton transfers during the DNA replication are far from equilibrium, a non-Poissonian distribution for tautomeric forms should be well founded. These results can be used to support the mechanism of the origin of spontaneous mutations in DNA based on concerted double proton transfers between complimentary nucleotide bases during the DNA replication instead of single proton transfers in a single base.

## Conclusion

In this work, we applied first-order autocatalysis to the Löwdin's mechanism of spontaneous mutation formation, in which concerted double proton transfers in DNA lead to the formation of tautomeric forms nucleotide bases during the DNA replication. The stochastic model results in a master-equation for the distribution function of tautomeric nucleotide bases, the stationary solution of which is a non-Poissonian function with the assumption that the processes of double proton transfers during DNA replication are far from equilibrium conditions. We suppose that these peculiarities of Löwdin's mechanism of spontaneous mutation formation should be taken into account in the discussion of the origin of spontaneous mutation formation. It is interesting to note the possibility of accumulation of point mutations locally on DNA due to the cooperation effect between tautomeric hydrogens. The cooperation effect should increase the order of autocatalytic reactions. Such fluctuations can be possible due to the U-negative effect (Anderson, 1975), which can lead to the formation of a solitary wave or a soliton on the chain at a certain distance between metastable proton pairs (Golo et al., 2001). The soliton can move along the chain and impacts on different biochemical processes, occurring in DNA, including its replication, proofreading and so on. At realization of such effect, the rate constants of the reactions (1) and (2) become the function of distance between the point mutations along the chain. This perspective of fluctuations will be considered in our future research.

## Data Availability Statement

All datasets generated for this study are included in the article/Supplementary material.

## Author Contributions

NT was the author of the idea about application of stochastic theory to spontaneous mutations in DNA and the main contributor of the manuscript. BO was the author of the idea about the possibility of soliton generation in DNA based on accumulation of metastable proton fluctuations.

### Conflict of Interest

The authors declare that the research was conducted in the absence of any commercial or financial relationships that could be construed as a potential conflict of interest.
